# Distribution of human papillomavirus in precancerous and cancerous cervical neoplasia in Tunisian women

**DOI:** 10.1186/s13027-021-00392-1

**Published:** 2021-07-16

**Authors:** Rahima Bel Haj Rhouma, Monia Ardhaoui, Emna El Fehri, Asma Marzougui, Thalja Laassili, Ikram Guizani, Med Samir Boubaker, Emna Ennaifer

**Affiliations:** 1 Department of Human and Experimental Pathology, Pasteur Institute of Tunis, University Tunis El Manar, Tunis, Tunisia; 2 Laboratory of Molecular Epidemiology and Infectious Diseases, Pasteur Institute of Tunis, University Tunis El Manar, Tunis, Tunisia

**Keywords:** Cervical neoplasia, Cervical cancer, Human papillomavirus, Genotype distribution

## Abstract

**Background:**

High-risk human papillomavirus (HR-HPV) are responsible for cervical cancer (CC) which represents the second most prevalent gynecological cancer among Tunisian women. Preventive strategies against CC are based on prophylactic vaccines that have not yet been implemented into the national vaccination program of Tunisia. Therefore, the present study aimed to investigate the HPV genotypes distribution in cervical neoplasia in Tunisian women in order to predict the impact of using current HPV vaccines on cancer prevention in Tunisia.

**Methods:**

A total of 200 formalin-fixed paraffin embedded biopsies were collected in our study. DNA was extracted using Qiagen Mini prep kit. DNA quality was controlled by Beta Globin PCR. Only positive samples for Beta Globin test were used. HPV detection was performed by a nested PCR using PYGMY and GP5+/6+ primers. Genotyping was performed by Reverse Line hybridization using 31 probes.

**Results:**

The mean age of participants was 38.97 years and 75% were over 30 years. Cervical neoplasia distribution according to age showed that CINII/CINIII was observed among women over 30 years old. All samples were positive for Beta Globin PCR. Overall HPV prevalence in cervical lesions was 83% (166/200). HPV was present in 65% of CINI, 82% of CINII/CINIII and 85% of CC. HR-HPV was statistically significantly associated with cervical intraepithelial neoplasia (p < 10^–3^). HR-HPV distribution according to lesion grade and cervical cancer showed that HPV16 and HPV18 were present in all lesions. For CINII/CINIII, HPV 35 (37.5%) was the most detected type, followed by HPV18 (33.3%) HPV 45 (28.5%) and HPV 16 (18.9%). HPV 45(57.5%), HPV 18 (53.3%) were the most detected in CC. HPV58, 59, 68 were only detected in CC and associated with HPV45, 18 and HPV16. HPV39, 31, 33, 52, 56 and HPV70 was associated only with CINI.

**Conclusions:**

Our findings can give useful information for vaccine implementation by helping the health policymakers to choose the most appropriate vaccine type in Tunisia.

## Background

Cervical cancer (CC) is the second most prevalent gynecological cancer in women worldwide [1, 2] with a global annual incidence of 569,847 in 2018, and 311,365 annual death rates [3]. In Northern Africa, the incidence of CC is 7652 cases with 5243 deaths annually [3]. Among Tunisian women, CC ranks second after breast for gynecological cancer [4], with an incidence of 3.8% (342 new cases in 2020) and about 200 deaths per year [5–7].

Pap smear screening has not been successful for preventing CC in Tunisian women. It seems necessary to offer new epidemiological considerations that could possibly include vaccine implementation and HPV testing in a national efficient preventive strategy.

It is well known that HPV is the necessary causal agent of CC [8]. About 201 different HPV types have been identified. More than 40 types infect the mucous membranes and have been classified into Low-risk (LR-HPV) and High-risk (HR-HPV) depending on their ability to induce malignant progression [9, 10]. Most HPV infections are cleared by the immune system. Cervical intraepithelial neoplasia (CIN) is a progressive pathophysiological process with two different pathways [11]. The first is a spontaneous regression of the cervical lesions. The second is the persistence of the infection with a HR-HPV that can progress to high grade intraepithelial neoplasia (CINII, CINIII) and CC in 8 to 12 years [10, 12, 13].

As HPV infection prevalence and genotype distribution is known to vary significantly in different countries and world regions [14, 15], their investigation in specific areas provides a scientific basis for the choice of appropriate measures and methods towards an efficient preventive and therapeutic strategy, which includes vaccine implementation and HPV testing. To our knowledge, prevalence and distribution of HPV types in different stages of cervical intraepithelial neoplasia has not yet been studied in Tunisia. This work aims to provide Tunisian empirical data that can be useful for an efficient management of cervical intra-epithelial lesions in Tunisian women.

## Methods

### Population study

This is a retrospective study including specimen of 200 women aged between 19 and 59 years with cervical intraepithelial neoplasia diagnosed on biopsies. Samples are paraffin embedded blocks that were collected between 2016 and 2019 in the department of pathology of Pasteur Institute of Tunis. The distribution of the cervical intraepithelial neoplasia according to the Bethesda system [16] was 82 CINI, 92 CINII/CINIII and 26 CC.

### DNA extraction

Paraffin blocks were cut into 10 µm thin preparations and added to a labeled tube. The rewashing step was performed by three washing baths in 800 μL of Xylen (incubation at 65 °C for 15 min) followed by four washings with 800 µL of ethanol with decreasing concentrations (100%–80%–60%–40%). DNA was then extracted by Qiagen Mini prep kit (Qiagen, CA, USA) according to the manufacturer’s instructions. DNA purity and concentration were measured by Nano-drop spectrophotometer.

The DNA quality was evaluated by a β-globin test using specific primers PC04/GH20. The β-globin PCR reaction was at a final volume of 25 µL and contained 50 ng/µL of DNA sample, 2.5 µL of 10X PCR buffer solution [670 mM Tris–HCl pH 8.8, 67 mM MgCl_2_, 167 mM (NH_4_)_2_SO_4_, 100 mM 2-mercaptoethanol], 1.5 µL of 10 mM dNTP mix, 1.25 µL of each primer (10 µM), 0.15 µL of Taq DNA polymerase (Thermo Scientific) and 17.6 µL of sterile distilled H_2_O. The cycling conditions for β-globin PCR were carried out with a first denaturation step at 95 °C for 4 min, followed by 35 cycles at 94 °C for 30 s, 40 °C for 30 s, 72 °C for 1 min and then a final extension step at 72 °C for 45 s.

### HPV DNA detection and typing

HPV detection was performed by a nested PCR using biotinylated PGMY09/11 primers for the first PCR and GP5+/GP6+ for the second PCR. PCR was performed with a positive control (plasmid carrying cloned HPV16 5GE/µL) and a negative control (PCR mixture without HPV DNA). Briefly, 50 µL mixture containing 3 mM MgCl_2_, 10 µmol of each primer, 1.5 mM of dNTP (dATP, dCTP, TTP, dGTP), 5 µL of supplied Buffer with the Taq DNA polymerase, 1 U of Taq DNA Polymerase, and 50 ng/µL of DNA preparation. The PCR amplification parameters were a 10 min initial denaturation at 94 °C, followed by 30 amplification cycles made of 30 s at 94 °C, 1 min at 50 °C and 1 min at 72 °C, and then a final extension step for 7 min at 72 °C. This reaction was followed by a nested PCR using 10 µL of PGMY PCR product in a reaction mixture containing 50 µmol of GP5+/6+ primers, 3 mM MgCl_2_, 1.5 mM each of the dNTP, 1 U of Taq DNA Polymerase and 5 µL of the buffer that was supplied with the Taq DNA polymerase. The program of reaction consists of a 10 min initial denaturation at 94 °C, followed by 40 amplification cycles made of 1 min at 94 °C, 2 min at 40 °C and 1.5 min at 72 °C, and a final extension step for 7 min at 72 °C.

Genotyping of positive samples was performed by Reverse Line Hybridization as described in the Human Papillomavirus Laboratory Manual published by the World Health Organization [17]. Briefly, 15 μL of denatured PCR products were allowed to hybridize with specific oligonucleotide probes for 31 HPV types (HPV6, 11, 16, 18, 26, 31, 33, 34, 35, 39, 40, 42, 44, 45, 51, 52, 53, 54, 55, 56, 57, 58, 59, 66, 68, 69, 70, 73, 82, 83, and 84) that were immobilized on a Biodyne C membrane using the Miniblotter MN45 (Miniblotter® 20SL, Immunetics®). The hybridized DNA was detected using Streptavidin HRP (BD Pharmingen™) and (3,3-diaminobenzidine (DAB, Abcam).

### Statistical study

Statistical analyses used the Statistical Package for the Social Sciences (SPSS) software version 20.0 (IBM, Somers, NY, USA). Pearson’s chi-square test was calculated to associate HPV detection and HPV genotypes with different variables. P values < 0.05 were considered statistically significant.

### Ethical considerations

The study was approved by the Ethical committee of Institut Pasteur de Tunis (2018/03/LAPHEIPT) and conducted with good clinical practice, ensuring confidentiality and anonymity.

## Results

### Target population

A total of 200 samples were included in this study. The mean age of participants was 38.97 years, and 75% (151/200) were over 30 years.

Cervical neoplasia distribution according to age showed that CINII/CINIII were observed in women over 30 years old (Table 1). No statistically significant association between age and lesions was found (p > 0.05).

**Table 1:** Intraepithelial cervical lesions distribution according to age group

	CINI	CINII/CINIII	CC	Total
	n (%)	n (%)	n (%)	
< 30	30 (61%)	16 (33%)	3 (6%)	49
30–39	36 (43%)	38 (46%)	9 (11%)	83
40–50	16 (27%)	32 (53%)	12 (20%)	60
> 50	0 (0%)	6 (75%)	2 (25%)	8
Total	82	92	26	200

*CINI* cervical intraepithelial neoplasia I, *CINII* cervical intraepithelial neoplasia grade II, *CINIII* cervical intraepithelial neoplasia grade III

### Overall HPV prevalence

All samples were positive for the β-globin test. HPV detection and typing were performed for 200 samples. HPV was detected in 166 samples leading to a global prevalence of 83%.

**Fig. 1:**
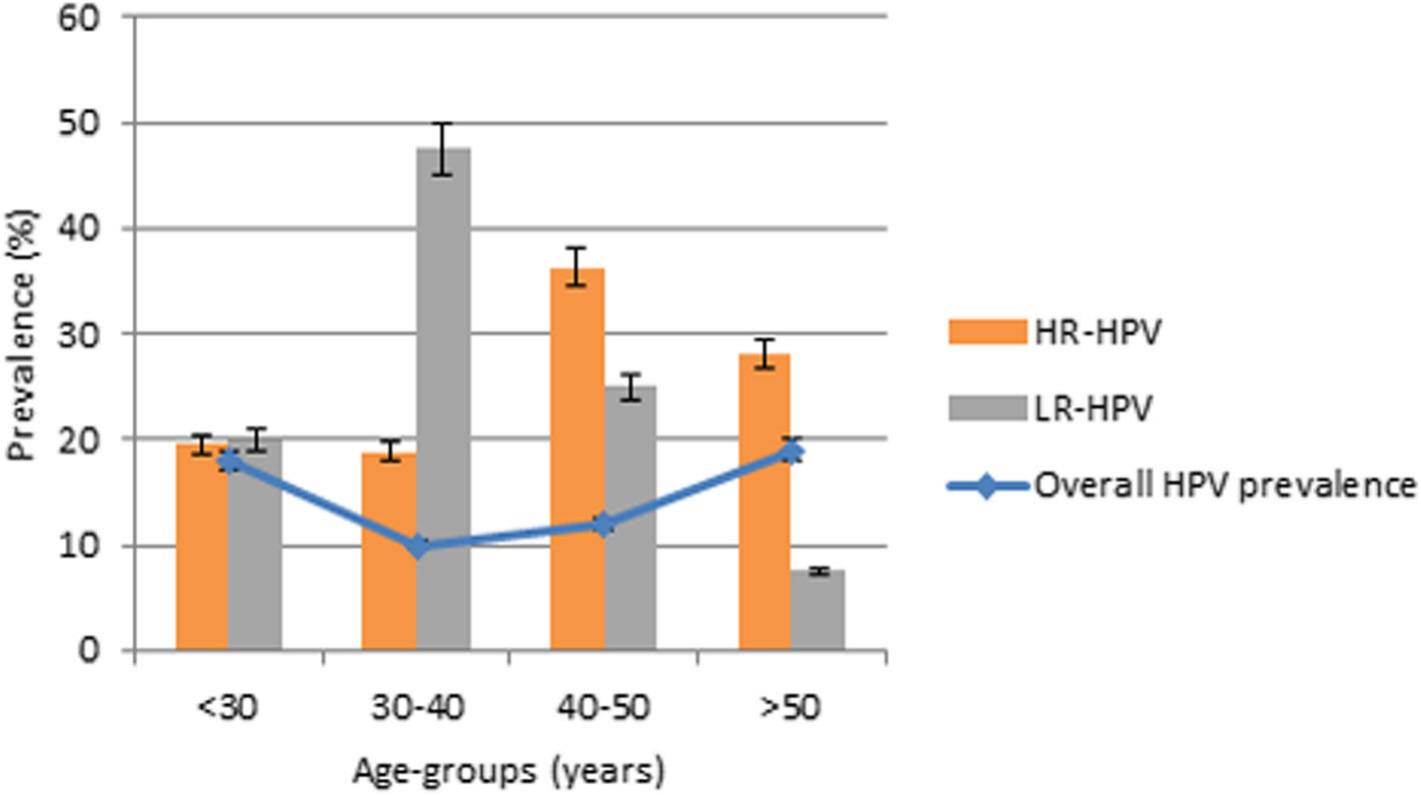
HPV prevalence distribution according to age groups. Error bars: percentage error 5%

The mean age of HPV positive women was 39.56 years. The most prevalent age group within HPV positive women was < 30 and > 50 (Fig. 1) without statistical significance (p > 0.05).

**Fig. 2:**
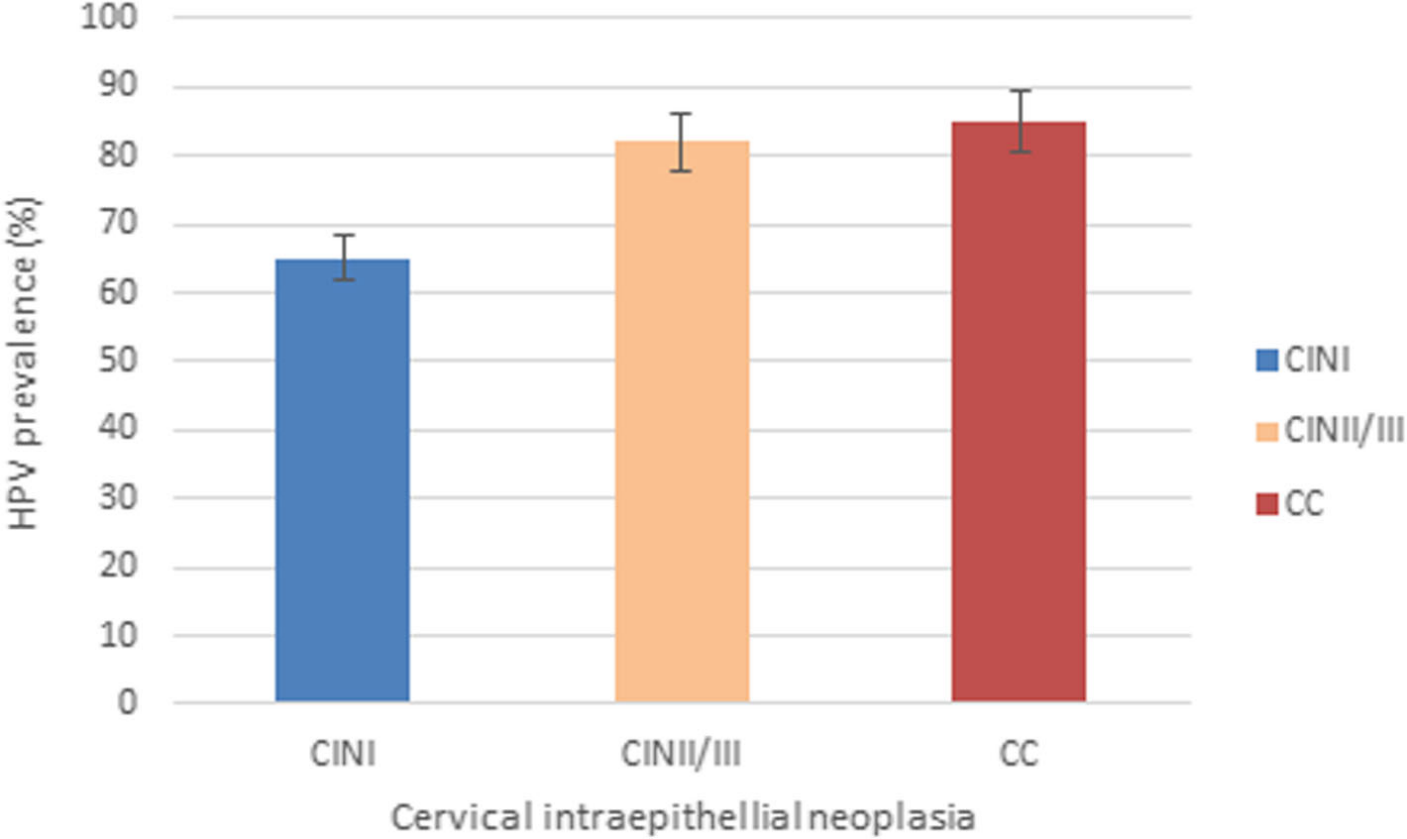
HPV prevalence according to the cytological statute. Error bars: percentage error 5%

HPV was present at the rate of 65% in CINI (53/82). For CINII/CIN III and CC HPV prevalence was respectively 82% (75/92) and 85% (22/26) (Fig. 2).

### HPV genotype distribution

A total of 22 genotypes was identified and were classified into HR-HPV types and LR-HPV.

**Fig. 3:**
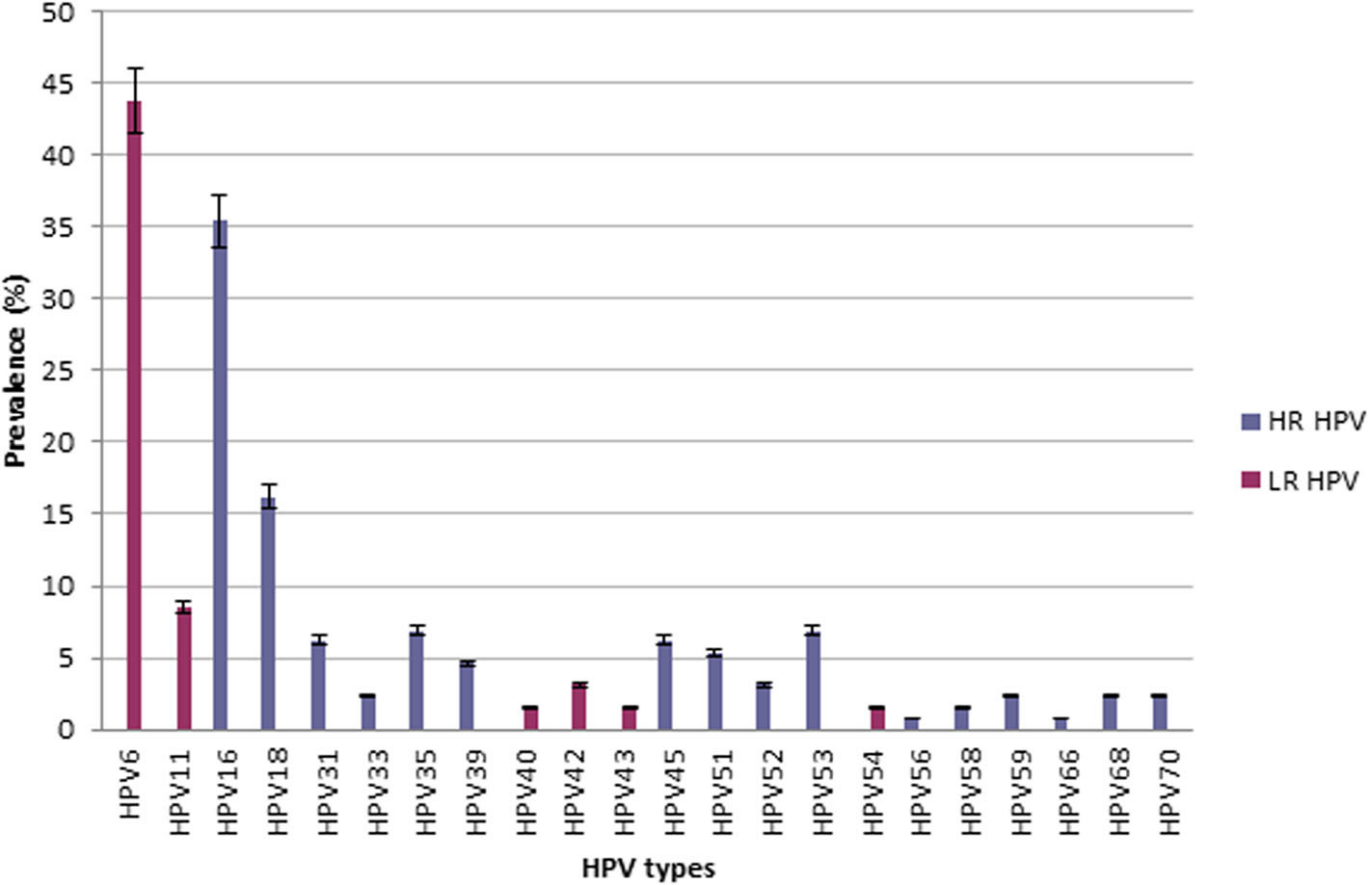
Distribution of HR-HPV and LR-HPV genotypes intraepithelial cervical neoplasia. Error bars: percentage error 5%

In this study, HR-HPV and LR-HPV prevalence were respectively 56% (112/200) and 37.5% (75/200). The most prevalent LR-HPV type was HPV 6 followed by HPV11, 42, 40, 43 and HPV 54. The predominant HR-HPV were HPV 16, 18, 53, 35, 45, 39, 51, 52, 33, 59, 68, 70, 66 and HPV 56 (Fig. 3).

Multiple and single infections were detected respectively in 44.5% (89/200) and 38.5% (77/200) of cases. No statistical association between infection type and age group or cervical intraepithelial neoplasia (Table 2).

**Table 2:** Infection type distribution according to age group and intraepithelial cervical neoplasia

	Multiple infection	Single infection	*P*
	n (%)	n (%)	
Age-group (years)			
< 30	19 (9.5%)	15 (7.5%)	0.8
30–39	32 (16%)	26 (13%)	
40–50	27 (13.5%)	25 (12.5%)	
> 50	11 (5.5%)	11 (5.5%)	
Cervical intraepithelial neoplasia			
CINI	30 (15%)	23 (11.5%)	0.16
CINII/CINIII	42 (21%)	30 (15%)	
CC	17 (8.5%)	5 (2.5%)	

*CINI* cervical intraepithelial neoplasia I, *CINII* cervical intraepithelial neoplasia II, *CINIII* cervical intraepithelial neoplasia grade III, *p* p value for the association between HPV type infection, age and cervical neoplasia

The association between age and HPV types showed that LR-HPVs and HR-HPVs were most prevalent among the age group (30–40) and (40–50), respectively (Fig. 1).

### HR-HPV types distribution according to cervical intraepithelial neoplasia

HR-HPV distribution according to cervical intraepithelial neoplasia showed that HPV 16 and HPV18 were present in all grades (Table 3). HPV16 and HPV18 were associated with HPV58, HPV59, HPV66 which were present only in CC. HPV 39, 31, 33, 52, 56, 68 and HPV70 were detected only in CINI (Table 3). For CINII/CINIII, HPV 35 (37.5%) was the most detected type, followed by HPV18 (33.3%), HPV 45 (28.5%) and HPV16 (18.9%). HPV 45 (57.5%), HPV 18 (53.3%) and HPV16 (24.3%) were the most detected in CC. HR-HPV was associated with cervical intraepithelial neoplasia (p < 10^–3^).

**Table 3:** Distribution of HR-HPV types according to cervical intraepithelial neoplasia

	CC	CINI	CINII/CINIII	Total
	n (%)	n (%)	n (%)	
HPV16	9 (24.3%)	21 (56.7%)	7 (18.9%)	37
HPV18	8 (53.3%)	2 (13.3%)	5 (33.3%)	15
HPV31	0 (0.0%)	7 (100.0%)	0 (0.0%)	7
HPV33	0 (0.0%)	2 (100.0%)	0 (0.0%)	2
HPV35	2 (25%)	3 (37.5%)	3 (37.5%)	8
HPV39	0 (0.0%)	5 (100.0%)	0 (0.0%)	5
HPV45	4 (57.5%)	1 (14.2%)	2 (28.5%)	7
HPV51	0 (0.0%)	5 (83.3%)	1 (16.6%)	6
HPV52	0 (0.0%)	3 (100.0%)	0 (0.0%)	3
HPV53	3 (37.5%)	5 (62.5%)	0 (0.0%)	8
HPV56	0 (0.0%)	1 (100.0%)	0 (0.0%)	1
HPV58	2 (100.0%)	0 (0.0%)	0 (0.0%)	2
HPV59	3 (100.0%)	0 (0.0%)	0 (0.0%)	3
HPV66	2 (50.0%)	2 (50.0%)	0 (0.0%)	4
HPV68	1 (100.0%)	0 (0.0%)	0 (0.0%)	1
HPV70	0 (0.0%)	3 (100.0%)	0 (0.0%)	3

*CINI* cervical intraepithelial neoplasia I, *CINII* cervical intraepithelial neoplasia II, *CINIII* cervical intraepithelial neoplasia III

## Discussion

Cervical cancer is a multistep disease and persistent infection with HR-HPV is the major cause of intraepithelial neoplasia and cervical cancer. An efficient preventive and therapeutic strategy including vaccine consideration and HPV testing needs to determine the HPV genotypes distribution in different cervical lesions. Previous Tunisian studies has been conducted on cervical cancer or women with normal pap smear [4, 18]. To our knowledge, there are few epidemiologic data concerning HPV genotypes in cervical neoplasia from Tunisia.

Our study provides results about HPV genotypes distribution in different stages of cervical lesions that could be useful for a national preventive strategy of CC and therapeutic algorithms for CIN including vaccine implementation and HPV testing.

We found that there are two age peaks of HPV infection prevalence in women with cervical neoplasia: less than 30 years old and over 50 years old. The infection rate during these ages was significantly higher compared to other age groups, suggesting a “U”-shaped infection. Our results are not in agreement with previously studies where authors reported that the highest peak was obtained for younger women (under 25 years old), then a decreasing trend with age was observed and another maximum peak around 50 years old [15, 19–23]. Many variables including geographic locations and population, demographic factors and possibly vaccination could be responsible for this difference in the prevalence of HPV-positive status and age trend modification.

In our population study, global HPV prevalence in cervical lesions was 83%. Our data showed that 65% of patients with CINI, 82% with CINII/CINIII and 85% with CC were HPV positive. These results are concordant with other studies conducted in Sousse governorate in the North-East of Tunisia which shows that overall HPV prevalence is 73.6% with 84% in CINI and 83.9% in CII/III [24]. Our results are also consistent with those reported worldwide [25, 26]. A study by Berraho and al. reported that HPV infection rate reached 92.5% in CC in Morocco [27]. The HPV positivity in Longnan-China women was 74.6% in CINI, 87.5% in CINII/CINIII and 89.05% in CC [28].

We can conclude that despite the small size of our subgroup, our results are in agreement with several reports. Our data confirm the correlation between HPV infection prevalence and the gravity of cervical intraepithelial neoplasia subgroup [23, 28, 29].

Of all HPVs positive samples, there were 43% of patients with multiple HPV types in all cervical neoplasia (CINI, CINII/III and CC). Even if our findings highlight the increasing of LR-HPV in (30–40) trend and HR-HPV in (40–50) trend, no statistically significant association between coinfection and age was found. These results are consistent with previous studies [19] reporting an age-specific prevalence of multiple HPV infections and a decreasing of multiple HR-HPV infection rates in (CINI, CINII/III and CC) compared to cervicitis.

In our series, HR-HPV was associated with cervical intraepithelial neoplasia (p < 10^–3^). The predominant HR-HPV was HPV 16, 18, 53, 35, 45, 39, 51, 52, 33, 59, 68, 70, 66, 56. HR-HPV were observed in lower age < 30-years group and mostly in women in the age group (40–50). In the study of Guardado-Estrada [30], authors reported that in CC Mexican patients, the first peak was found in young women under 35 years, the second peak was at 61–65 years and the mean ages of the patients with single infection with HPV16, HPV 18, HPV 45 and HPV 39 were at least 5 years lower compared to the patients with single infection or double infection with other HPV types and HPV 16, 18, 45 and 39 [30]. Aro and collaborators [19] reported that in CINII/CINIII, HPV16 and HPV18 are more common in younger women, below 30 years old and over 45 years old [19].

CINI may appear within 4 months after HPV infection and if associated with certain HPV genotypes, could progress to CINII, CINIII and to cervical cancer. Assessing persistent HR-HPV genotypes among CINI is therefore suitable to identify women with risk of progression. Our results indicate that the most frequent HPV genotypes in CINI were HPV31, 33, 39, 52, 56, 68, 70, 51 and HPV 53. The meta-analysis of Guan et al. [31] reported that HPV 35, 39, 51, 56 and 68 were present in low and high grade lesions but were not frequent in CC which is in favor of the low carcinogenic potential of this types.

In this study the most prevalent genotypes in CINII/CINIII were HPV 35, 18, 45 and HPV16. In CC, HPV45, 18, 35 and HPV16 were predominant. HPV 16 and HPV18 were detected in all lesions. Our results are concordant with the meta-analysis of Clifford et al., highlighting that in the fifteen high risk HPV genotypes, HPV16 and 18 were found in approximately 70% of cervical cancer worldwide [32]. We also demonstrated that HPV16, 18 and 45 were detected in CC and that HPV45 was mostly present in CINII/CINIII and CC.

In fact, HPV 45 seems to be more frequent in Africa and less in European, American, and Asian populations [26, 28, 31]. In Europe, HPV16 and HPV18 are the most common types. HPV 58 is the most prevalent in Asia. In Saudi Arabia, the most common genotypes in CC were: HPV16, 18, 31, 45, 56, 59 and HPV73 [33]. In a worldwide meta-analysis of over 115,000 HPV-positive women HPV16, HPV18 were the most prevalent HPV types in cervical lesions while HPV52 and HPV58 were most prevalent in East Asian women [14]. Another study in India described HPV16, 18 and 58 as the most common types [15].

In this study, we reported the HPV genotypes distribution in different cervical intraepithelial neoplasia grades which can guide the use of adequate vaccine in Tunisia. Nowadays, three HPV vaccines are available and commercialized: the bivalent vaccine includes the HR-HPV genotypes 16 and 18 found in 70% of CCs. The quadrivalent vaccine comprises, in addition to the above-mentioned genotypes, the LR-HPV HPV-6 and -11 that cause 90% of genital warts and finally, the recent nano-valent vaccine includes the HR-HPV genotypes 16, 18, 31, 33, 45, 52, and 58 and the low-risk HPV genotypes 6 and 11 [5]. This latter seems to include HPV genotypes reported in our study. Interestingly, Joura et al. [34] reported that administration of the nano-valent vaccine before exposure to described genotypes, can achieve 90% of cervical cancer protection [34].

Even if our results are sound, the present study has the limitation of being focused only in one sampling center (Institut Pasteur de Tunis) and warranted to be extended to a group of women representatives of all the region of Grand Tunis.

## Conclusions

In Tunisia, data about HPV distribution in intraepithelial cervical neoplasia are still lacking. Our findings support that HR-HPV prevalence increases from CINI to CINII/CINIII and CC. HPV 35, 18, 45, 16 are the predominant HR-HPV in women with high grade intraepithelial cervical neoplasia CINII/CINIII. The results of the present study can help the health policymakers to make a decision on the introduction of HPV vaccines and to choose the adequate vaccine type to be included in Tunisian national vaccination program.

## Data Availability

All data generated or analyzed during this study are included in this published article.

## Abbreviations

HPV: Human papillomavirus
HR: High risk
LR: Low risk
CINI: Cervical intraepithelial neoplasia I
CIN II: Cervical intraepithelial neoplasia II
CINIII: Cervical intraepithelial neoplasia III
CC: Cervical cancer
